# Production and Analysis of the Physicochemical Properties of the Pyrolytic Oil Obtained from Pyrolysis of Different Thermoplastics and Plastic Mixtures

**DOI:** 10.3390/molecules27103287

**Published:** 2022-05-20

**Authors:** Paul Palmay, Carla Haro, Iván Huacho, Diego Barzallo, Joan Carles Bruno

**Affiliations:** 1 Escuela Superior Politécnica de Chimborazo ESPOCH, Panamericana Sur Km 1 1/2, Riobamba 060155, Ecuador; carlav.haro@espoch.edu.ec (C.H.); ivan.huacho@espoch.edu.ec (I.H.); 2 Facultad Ciencias e Ingeniería, Universidad Estatal de Milagro, Milagro 091050, Ecuador; dbarzallog@unemi.edu.ec; 3 Environmental Analytical Chemistry Group, University of the Balearic Islands, Cra. Valldemossa Km 7.5, 07122 Palma de Mallorca, Spain; 4 Department of Mechanical Engineering, Universitat Rovira i Virgili, Avenida Paisos Catalans, 26, 43007 Tarragona, Spain; juancarlos.bruno@urv.cat

**Keywords:** pyrolysis, plastics waste, bio fuels

## Abstract

The constant search for the proper management of non-degradable waste in conjunction with the circular economy makes the thermal pyrolysis of plastics an important technique for obtaining products with industrial interest. The present study aims to produce pyrolytic oil from thermoplastics and their different mixtures in order to determine the best performance between these and different mixtures, as well as to characterize the liquid fraction obtained to analyze its use based on said properties. This was carried out in a batch type reactor at a temperature of 400 °C for both individual plastics and their mixtures, from which the yields of the different fractions are obtained. The liquid fraction of interest is characterized by gas chromatography and its properties are characterized by ASTM standards. The product of the pyrolysis of mixtures of 75% polystyrene and 25% polypropylene presents a yield of 82%, being the highest, with a viscosity of 1.12 cSt and a calorific power of 42.5 MJ/kg, which has a composition of compounds of carbon chains ranging between C6 and C20, for which it is proposed as a good additive agent to conventional fuels for industrial use.

## 1. Introduction

Currently, the great worldwide demand for the use of fossil fuels such as gasoline or diesel has caused the large fluctuations in their marketing prices, which has drastically affected the economies of many countries, especially developing countries. In this context, the aim is to promote a circular economy of plastic waste to generate compounds with characteristics that help to replace conventional fuels totally or partially through the use and optimization of relatively new technologies such as pyrolysis [1,2,3]. Thermal pyrolysis is a versatile process for the final disposal of different urban plastic waste products such as polypropylene (PP), polystyrene (PS), polyethylene (PE), polyethylene terephthalate (PET), or polyvinyl chloride (PVC) [4], which entail endothermic reactions at high temperatures, low pressures, and/or with the generation of a vacuum to obtain mainly biofuels [5], thus being a great alternative to closing the circle of the circular economy by reintroducing high-quality recycled plastic into the economy. Three fractions are obtained from the pyrolysis process: a liquid fraction (called pyrolytic oil: the desired main product) made up of a mixture of hydrocarbons without the presence of waxes, with molecules whose molecular weight is distributed between C6 to C28, and with a caloric value greater than 40 MJ/kg; a gaseous fraction (rich in organic vapors); and a solid fraction (from the decomposition of macromolecules in the absence of oxygen). In addition, this technology provides a great sustainable alternative in line with the principles of Green Chemistry since no additional chemical agent is needed and it drastically reduces the plastic waste generated.

Regarding the performance of the pyrolytic process, values of up to 95% (m/m) have been obtained, as well as values in its physicochemical properties such as viscosity of 3 mm^2^/s, a flash point of 30 °C, and a caloric value close to 42 MJ/kg similar to conventional diesel, thus obtaining very attractive compounds for use [6,7,8]. The properties of the main bioproduct obtained will depend largely on the compounds that make up the mixture of hydrocarbons from thermal degradation, the type of plastic, temperature, heating rate, retention time, and other process variables that can improve the efficiency, quantity, and consistency of the liquid oil [9]. The type of reactor used is of importance in the degradation process because according to the type of equipment the heat and mass transfer improves the production of light fractions. There are different technologies to carry out the pyrolysis reaction, among the most important is the scraw reactor. The pyrolysis occurs as the plastic residue passes through the hot zones and is particularly affected by the residence time of the material and the heating temperature, and its performance can be improved by varying the screw speed. It works with a mixture of plastic residue and oil resulting from the pyrolysis itself. A stirred tank reactor facilitates the handling of viscous fluids with a high generation of liquid and gaseous fractions and has in its structure an agitation system that homogenizes the raw material to uniformly decompose all the material contained in the reactor, increasing its performance. A fixed bed reactor has a low thermal conductivity for plastics especially in large reactors causing zones of different temperatures that affect pyrolysis. The fluidized bed reactor is one of the most widely used reactors at the industrial level due to its ease of heat transfer to the entire surface of the plastic throughout the reactor, increasing the product yield. This type of reactor uses temperature as the only means of chemical activation for a variety of catalysts. It is used for the decomposition of polystyrene, polypropylene, polymethylmethacrylate, mixed plastic polymers, rubbers, synthetic lubricants, and minerals [10,11,12]. On the other hand, most investigations carry out pyrolysis in discontinuous reactors to analyze the incidence on the yield and the aforementioned properties, at temperatures close to 400 °C, which can be estimated for individual samples by means of thermogravimetry determining the maximum degradation temperature for each plastic [13,14]. In the pyrolysis of PE waste, HDPE is the waste that is obtained in the greatest quantity with a yield of the liquid fraction greater than 50%, however, its composition presents waxy compounds with a high molecular weight, which generates compounds that, when below room temperature, tend to solidify or generate a synergy with little liquid and a greater presence of gums and waxes, with which for its use it must be subjected to a distillation process, thus reducing the percentage of usable product [15,16,17]. Another polyolefin present in waste is PP, which generates a significant gaseous fraction due to the degradation mechanism suffered by this structure through a breakdown mainly of the side chains, thus generating liquid fractions with yields close to 45% and a gaseous fraction higher than 30%. Regarding the pyrolysis of polystyrene, this process is usually oriented towards the depolymerization of the macromolecule, since by having an aromatic ring, it will mainly break the main chain and not the aromatic ring, by which the distribution of the products will be oriented towards the production of styrene, benzene, toluene, or compounds with several rings, which yield a liquid with a low viscosity and a high calorific power. The yields obtained with this raw material are high, close to 80%, with a fairly little gaseous generation compared to other plastics [18,19]. Likewise, the pyrolysis of PET provides a recovery percentage of the liquid fraction at the time of thermal degradation that is very low compared to other thermoplastics [7,20], in addition to the fact that this type of plastic is widely used in mechanical recycling [21,22]. Similarly, the pyrolysis of PVC, due to the fact that the pyrolytic products of this plastic contain hydrochloric acid, which can be corrosive for the equipment and its accessories, and toxic in the case of leaks, is not investigated, therefore other treatments are used for these residues [23].

Therefore, pyrolysis is an important plastic waste management process due to its generation of value-added products for industrial use. It can be carried out at different temperatures depending on the heating rate; however, to minimize energy consumption, studies must be carried out to determine the optimum temperature to produce products. Although the pyrolysis process can be carried out on any plastic waste, there is little information about specific mixtures and the characteristics of the pyrolytic oil obtained. Therefore, the present study aims to propose experimentation with the pyrolysis of thermoplastic residues in a temperature range estimated by thermogravimetric analysis to determine the temperature that generates the highest and best liquid product for the subsequent pyrolysis for the mixtures of said plastics. Additionally, this study presents a comparative analysis of the properties of the product obtained with the prospect of its use as a conventional fuel additive.

## 2. Materials and Methods

### 2.1. Sampling

The sampling of post-consumer plastics was carried out completely randomly, collecting 5 kg per day of all the plastics that arrive at the garbage dump in the city of Riobamba, Ecuador (around 250,000 inhabitants) for ten days for three months. On the first month, the first ten days were sampled, the second month the intermediate days, and on the third month the last ten days, thus creating a sample that simulates all the plastic waste production scenarios throughout the month. Subsequently, these residues are crushed to an approximate size of 1 cm and washed with a 1% NaOH solution to eliminate residues and gums that were present. The classified plastic waste has been characterized by Fourier Transform Infrared Spectroscopy (FTIR) using a JASCO FT/IR-4100 spectrometer. The method used has been executed with the Spectra Analysis program, which performs the data acquisition, treatment, and provides a numerical value based on the height or area of the peak in a working scan range of 4000 to 550 cm^−1^.

### 2.2. Pyrolysis Conditions

**Figure 1:**
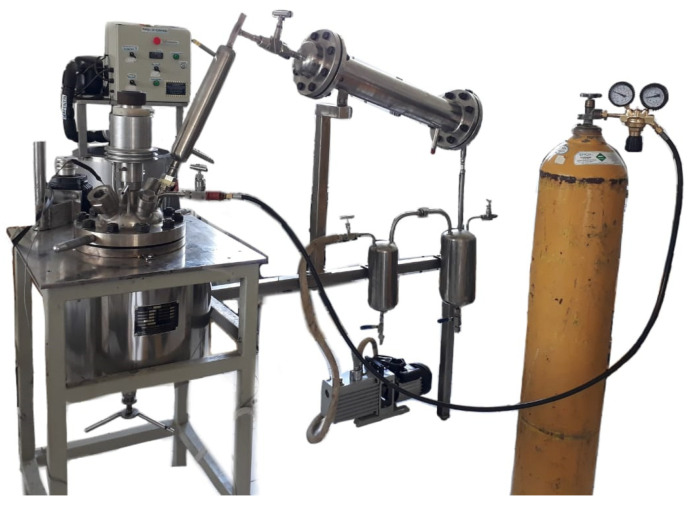
Scheme of the reaction unit.

In previous work by the authors, through thermogravimetric analysis, the maximum range of the degradation temperature was identified. These data were used to identify the pyrolysis temperature range (350–450 °C). The pyrolysis experiments shown in Figure 1 were carried out in a stainless steel batch reactor with a capacity of 5 L, model GSH-5.0L from the company Weihai Global Chemical Machinery, Shandong, China, with a built-in stirrer and a condensed oil collector. The reactor was coupled to a cooling system to provide a condensation temperature of 10 °C, in which, for each test, 1000 g of post-consumer plastic was fed, and then subjected to the operating conditions presented in Table 1.

It is worth mentioning that for PP, PS, and PE plastics, pyrolysis experiments were carried out in a temperature range from 350 °C to 450 °C to obtain the optimum temperature with a higher yield of the liquid fraction. For the mixture of plastics with the previous results, the working temperature was established and the yield and quality of the liquid fraction obtained at different mixing percentages were analyzed. Pyrolysis of PVC is not performed because the process generates hydrochloric acid which is toxic and corrosive to the reactor. For each experiment, three repetitions are carried out to obtain reliable data with a minimum deviation of results.

### 2.3. Liquid Fraction

A temperature range is established for PP, PS, and PE according to the analysis of its maximum degradation temperature determined in the thermogravimetric analysis (TGA) by using a 1 STAR System thermogravimetric analyzer (Mettler Toledo, Columbus, OH, USA) at a heating rate of 10 °C min^−1^, with a nitrogen atmosphere and at a flow rate of 20 mL min^−1^. However, the pyrolysis is not carried out with PVC because it can produce hydrochloric acid and neither with PET because due to their low performance of their products owing to its structure. From pyrolysis, the liquid and solid fractions are collected after each test to be weighed, while the gaseous fraction is obtained from the global mass balance. Furthermore, all experiments were performed in triplicate.

(1) $$\%\;liq=\frac{m_{líquid}}{m_{plastic}}\times 100\;$$

(2) $$\%\;sol=\frac{m_{líquid}}{m_{plastic}}\times 100\;$$

(3) % gas=100−%liq−%sol

The condensable compounds were recovered in the main separator, stored in amber bottles, and refrigerated at < 20 °C for their subsequent chemical and physicochemical characterizations.

### 2.4. Characterization

The characterization of the obtained mixtures was analyzed by means of gas chromatography coupled with a mass spectrum (GC-MS) to carry out its quantification (chemical mass composition), while the physicochemical properties were carried out by means of the guide of the norms that are presented in Table 2.

### 2.5. Plastics Mix

The process of the pyrolysis of the mixtures of PS, PP, and PET was proposed, and the combinations were labeled M1, M2, and M3 with the compositions 25% PP + 25% PS + 50% PET, 50% PP + 25% PS + 25% PET, and 25% PP + 50% PS + 25% PET, respectively. The labeling of the mixtures of PS, PP, and PE was proposed, having three mixtures called M4, M5, and M6 with the compositions: 50% PP + 25% PS + 25% PE, 50% PE + 25% PS + 25% PP, and 50% PS + 25% PE + 25% PP, respectively. According to the experimentation carried out, the pyrolysis process of PS and PP present liquid fractions rich in compounds similar to conventional fuels, and under the experimental conditions they did not present the presence of waxes or gums as the pyrolysis of PE or PET presented, which is why we carried out the pyrolysis of PS and PP mixtures having three mixtures called M7, M8, and M9 with the compositions (50% PP + 50% PS), (75% PS + 25% PP), and (25% PS + 75% PP), respectively.

## 3. Results

### 3.1. Characterization of Plastics Waste

**Figure 2:**
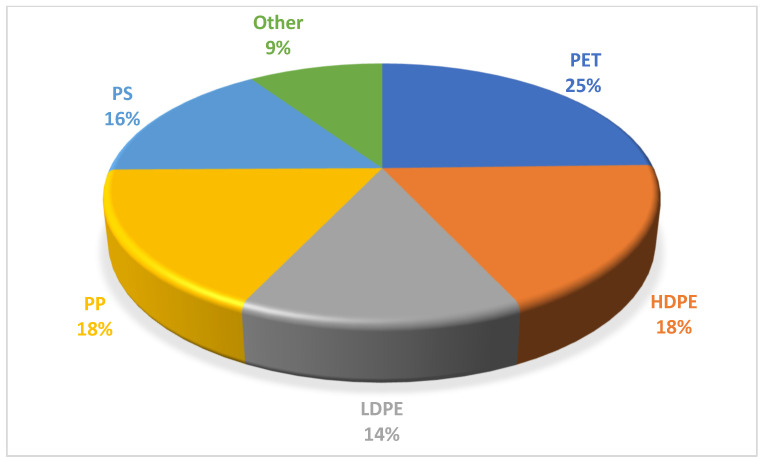
Composition of plastic waste.

The final sample was a composite sample with a final weight of 45 kg. Figure 2 shows the total percentages of each plastic present in the residues by FTIR spectroscopy.

### 3.2. Pyrolysis of Polypropylene (PP)

**Figure 3:**
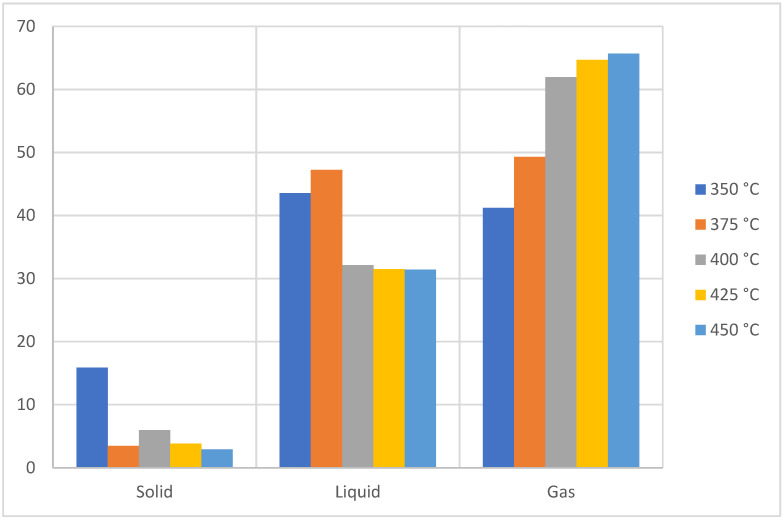
Yield of fractions, pyrolysis of PP.

Figure 3 shows the influence of temperature on the yield of the pyrolysis products, where it is evident that the formation of solid residues is favored at low temperatures (≤350 °C), while at high temperatures (≈450 °C) gaseous products are favored since the fractionation of the macromolecule results in obtaining low molecular weight molecules by increasing the temperature of the process [24]. As for the liquid products at low temperatures, they present a percentage of liquids close to 50% that, when the temperature increases, suffer a decrease, and in addition, the pyrolytic oils of the condensable phase visually appear as an oily fluid with a penetrating odor and a brown coloration at temperatures higher than 400 °C, while at 300 °C and 350 °C they appear as a higher viscosity fluid which at room temperature forms precipitates with a waxy appearance and an amber color [25]. Through the analysis of the variance between groups, a significant difference was determined between the temperatures of 350 °C, 375 °C, and 400 °C, while for temperatures of 400 °C, 425 °C, and 450 °C no statistical difference can be seen.

**Figure 4:**
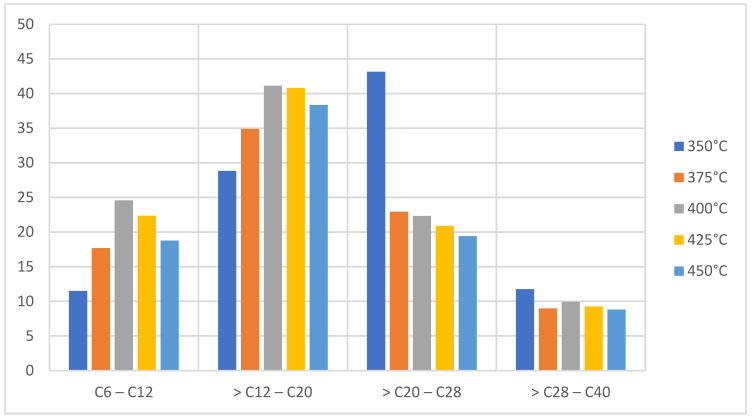
Mass chromatography of PP pyrolysis products.

On the other hand, taking into account that the gasoline/naphtha fraction is between C6 to C12, the diesel/kerosene fraction is between C12 to C20, and the semi-heavy fraction/oils are from C20 to C40. Figure 4 shows that at temperatures of 350 °C and 375 °C there is production of the liquid fraction greater than 40% with a content of semi-heavy hydrocarbons greater than 50%, which after cooling causes the oils from high molecular weight to form waxes of a brown coloration. At a temperature of 400 °C it presents a yield of 32% with a gasoline and diesel content greater than 60%, and a mixture that appears as a dark brown oily fluid with a characteristic pungent odor [9,26].

### 3.3. Pyrolysis of Polystyrene (PS)

**Figure 5:**
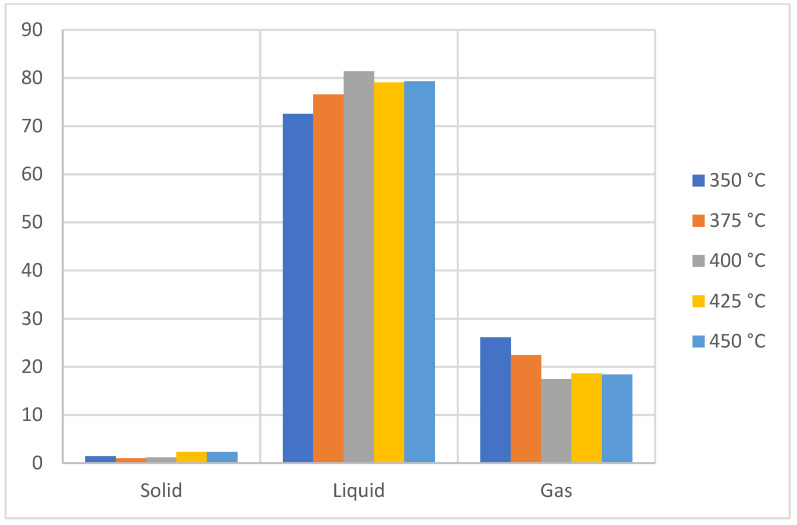
Yield of fractions, PS pyrolysis.

Figure 5 shows the three fractions obtained at different temperatures within the PS degradation range, which is very similar to PP, since the gaseous fraction increases as the temperature of the reactor increases. This behavior is maintained up to 425 °C, where, as at 450 °C, a constant behavior is observed in the generation of the fractions [26,27], and this is due to the fact that high temperatures cause the C-C bonds to have a greater breakage which generates lighter short-chain hydrocarbons [27,28] rich in aromatic hydrocarbons since the random breakage in the pyrolysis process of the main chain affects the aromatic chain to a lesser extent than the base structure of the macromolecule [29].

**Figure 6:**
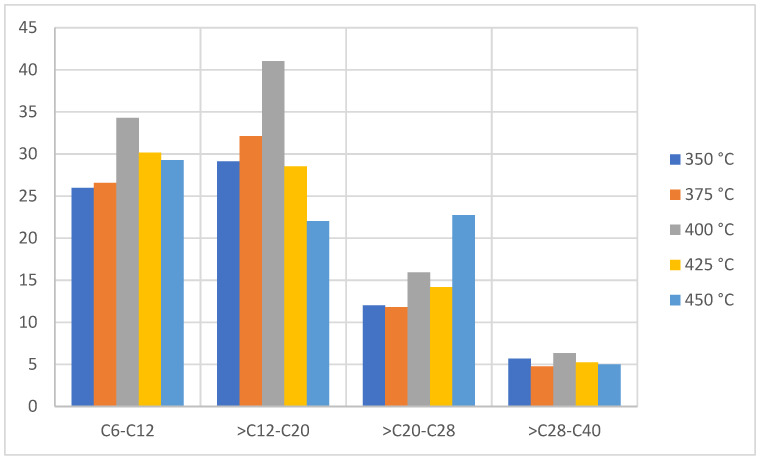
Mass chromatography of PS pyrolysis products.

Figure 6 shows that the pyrolytic products of this type of plastic tend to generate light components at a fraction greater than 70% with the presence of 5% of compounds with a molecular weight greater than C28 and with the orientation of the aromatic compounds. Additionally, the distribution according to the number of carbons of the products obtained with respect to temperature is observed, which shows an increase in compounds between C6 to C20 at 350 °C and a decrease at temperatures greater than 400 °C, since after reaching the maximum degradation temperature or close to it, secondary reactions occur between the radicals present in the equilibrium such as polyaromatic formation reactions, which decreases the yield of light oils [30,31]. In previous studies, an increase in the generation of styrene at higher temperatures [32], which is observed and is clearly pronounced in the case of compact polystyrene due to its crystalline structure, was represented in the high levels of hydrocarbons between C6 to C20, such as styrene, benzene, and toluene, which are potentially recoverable substances for industrial applications [33]. In short, the production of the liquid fraction for PS is favored at temperatures close to 400 °C with a fairly large composition of (light) aromatic compounds.

### 3.4. Pyrolysis of Polyethylene (PE)

**Figure 7:**
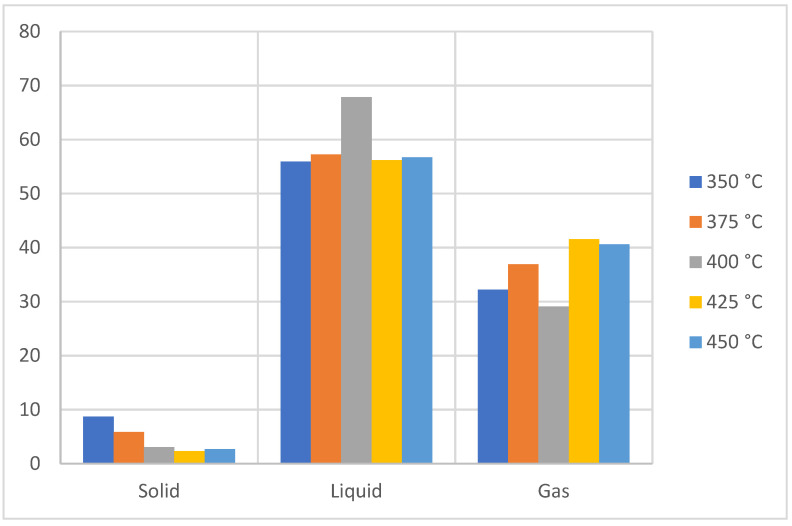
Yield of fractions, PE pyrolysis.

Figure 7 shows the yield of the products from the liquid fraction of the pyrolysis of the polyethylene plastic waste, where the temperature influences the liquid fraction, reaching its highest yield at a temperature of 400 °C with 68%. However, due to the characteristics of the process in the degradation of the linear structure of polyethylene, the mixture of hydrocarbons obtained has an oily appearance that at room temperature appears as brownish wax with a strong odor. In addition, as the pyrolysis temperature increases, the generation of the gaseous fraction increases due to the generation of light compounds from the breaking of the terminal bonds, which are easy to pyrolyze due to the very structure of PE [34,35], which increases from 11.85% to 350 °C to more than 40% at a temperature of 400 °C. On the other hand, the solid fraction decreases with the increase in temperature of 32.22% (*w*/*w*) at 350 °C, decreasing by 2.70% (*w*/*w*) at 450 °C. It should be noted that the liquid fraction at different temperatures shows the presence of waxes and gums, in accordance with what was stated by other authors [36,37].

**Figure 8:**
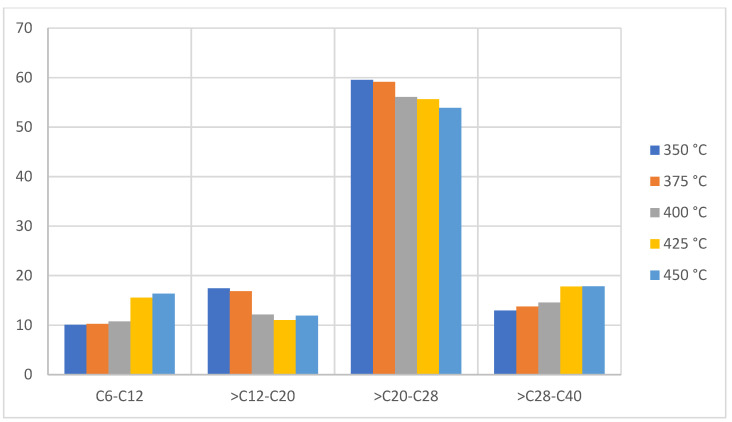
Mass chromatography of PE pyrolysis products.

A difference can be observed in the performance of the liquid fraction obtained between PP and PE, a difference that is also presented by the author of [8] in his study where he analyzes the pyrolysis of individual plastics and pure plastics. The values can be attributed to the methyl radical of the PP, since in the breaking of the bonds the radical will guide the formation of short gaseous chains [36,37]. In Figure 8, the most abundant hydrocarbon compounds in the analyzed sample are formed by carbons >C12–C28, which indicate that the liquid/wax fraction is rich in diesel-range hydrocarbons [38]. Therefore, if the product is fractionated in a distillation column, it would be possible to obtain mostly diesel. Likewise, it turns out to be a petrochemical rich in paraffins, naphthenes, and olefins, without the presence of benzene derivatives or conjugated aromatic structures, which can be used as a raw material to make virgin plastic or refined fuels. It is worth mentioning that the production of the liquid fraction benefits from the presence of low density polyethylene structures, and this suggests that the PE that has branches in the polymer skeleton (LDPE) benefits from the production of liquid hydrocarbons more than the linear chain PE (HDPE).

### 3.5. Plastics Mix

**Figure 9:**
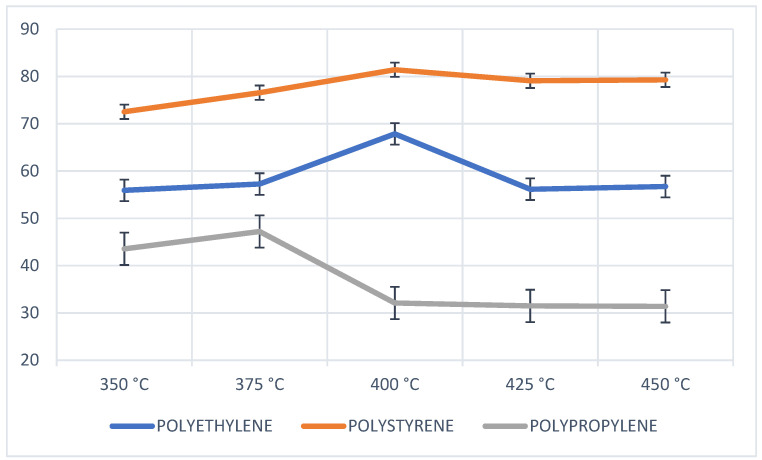
Liquid fraction of pyrolysis of plastics (PE, PS and PP).

The results of the pyrolysis of the individual PP, PS, and PE wastes show a high liquid fraction (PS and PE) and no wax content (PP) at a temperature of 400 °C, as shown in Figure 9, thus performing pyrolysis of the mixtures at this temperature.

#### 3.5.1. Polypropylene, Polystyrene and Polyethylene Terephthalate (PP + PS + PET)

**Figure 10:**
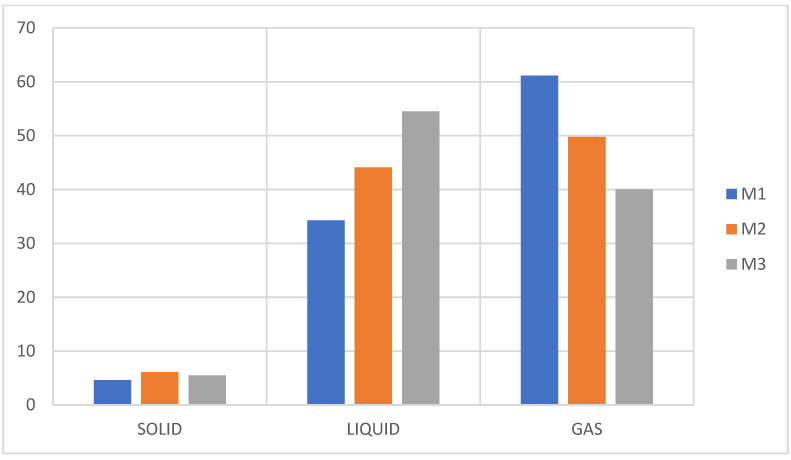
Yield of fractions, pyrolysis of M1, M2, and M3.

The results of the experimentation are presented in Figure 10.

The liquid fraction of the pyrolysis of these mixtures presented a different consistency and coloration than the liquid fraction of the pure plastics, having a yellowish-brown color, an oily appearance with an intense odor characteristic of the aromatic structure in the M3 sample, and a paraffin odor in the M1 sample due to the presence of more polystyrene in the first and the presence of more polypropylene in the second. This coloration is attributed to the presence of PET, which generates the production of branched compounds that include radicals with the presence of oxygen in the liquid oil produced [9].

The liquid fraction is benefits from the presence of a higher percentage of PS while the gaseous fraction is benefits from the presence of PET, being a midpoint in the mixture with a higher percentage of PP. Additionally, the presence of PP together with PS in greater quantity generates styrene isomers, which can be seen in samples M2 and M3, and tend to have a “lighter” coloration and the characteristic odor of aromatic compounds. As for sample M3, it presented a liquid fraction yield of 54%, differing from M2 by approximately 10%, and by M1 by 20%, being the mixture with the highest liquid fraction without the presence of waxes and coloration, and brown unlike M2 and M1.

#### 3.5.2. Polypropylene, Polystyrene and Polyethylene (PP + PS + PE)

**Figure 11:**
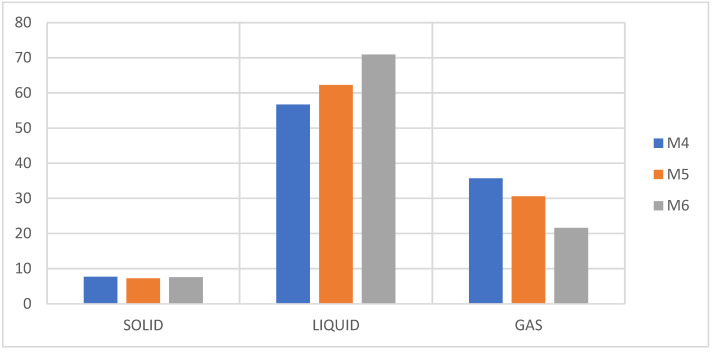
Yield of fractions, pyrolysis of M4, M5, M6.

The results of the experimentation are presented in Figure 11.

**Figure 12:**
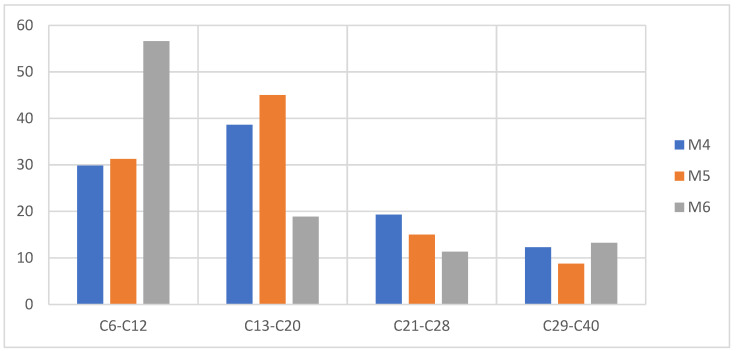
Mass chromatography of PS pyrolysis products, pyrolysis of M4, M5, M6.

Like the mixtures of PS + PP + PET, these samples present waxes and a brown coloration at a higher percentage of PE, and this occurs because the polyolefin of the PE in its pyrolysis generates compounds with a higher carbon number (>C20), while the presence of a higher percentage of PS generates a liquid fraction close to 70%, thus significantly reducing the presence of waxes, which favors the production of the aroma of aromatic compounds, as can be seen in Figure 12.

#### 3.5.3. Polypropylene and Polystyrene (PP + PS)

**Figure 13:**
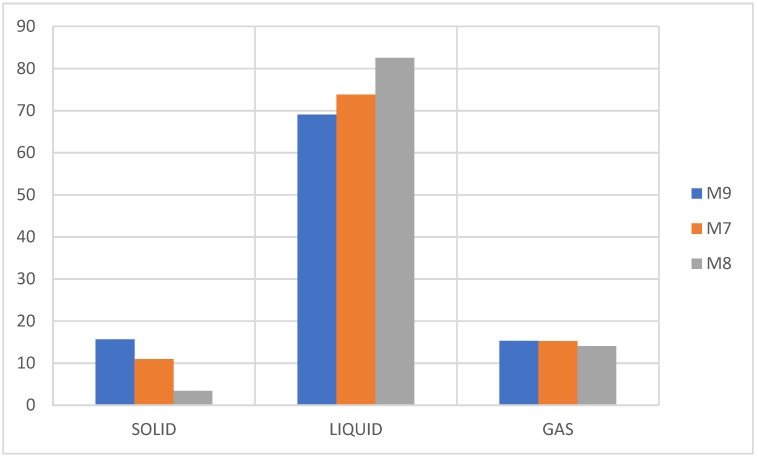
Yield of fractions, pyrolysis of M7, M8, M9.

Figure 13 shows that the efficiency of the liquid fraction is directly related to the amount of polystyrene, corresponding to the high fraction that is obtained when only PS is pyrolyzed, while in the solid fraction in all three cases coke is obtained showing a complete pyrolysis. The same one that presents an increase as the percentage of PP present in the mixture increases, attributable to the reaction mechanism of the thermal pyrolysis of the PP that occurs via the initial degradation of the ends of the chain generating chain structure cuts and/or carbonization of the plastic [7,9]. In the case of the gaseous fraction, there is no significant difference in any of the mixtures.

**Figure 14:**
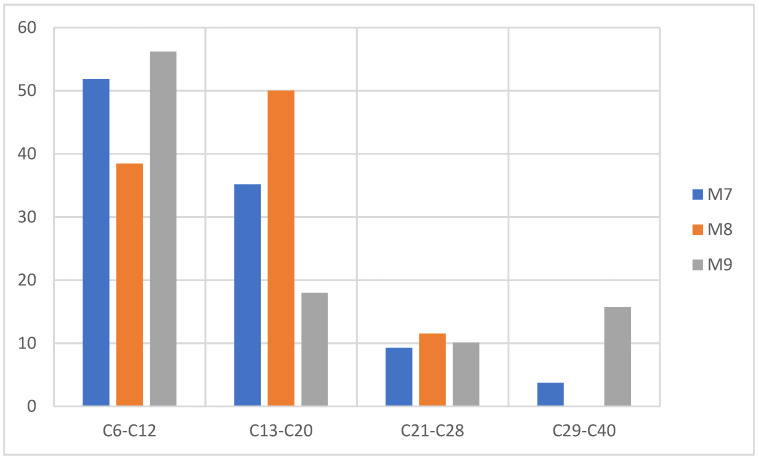
Mass chromatography of PS pyrolysis products, pyrolysis of M7, M8, M9.

Due to the mechanism that follows the degradation (thermal pyrolysis) of PS, there is the presence of a carbocation that is not very stable in its structure at the moment of the generation of the radical in the initiation stage of the reaction, and this is due to the controlled addition of temperature to any heating rate. This is the reason why the rupture in the polymer structure occurs mainly in the main chain (depolymerization process), generating mainly aromatic compounds of one ring or several, thus obtaining a high liquid fraction [30,33,39]. The conjunction with the PP at the time of pyrolysis generates aromatic compounds linked to short linear and branched chains provided by the PP, which causes a distribution of the compounds between C6 and C20 as can be seen in Figure 14, where it is evident that at an even higher percentage of PS the liquid fraction is higher and its distribution is oriented in short chains between C6 and C20.

### 3.6. Analysis of the Performance of the Fractions

**Figure 15:**
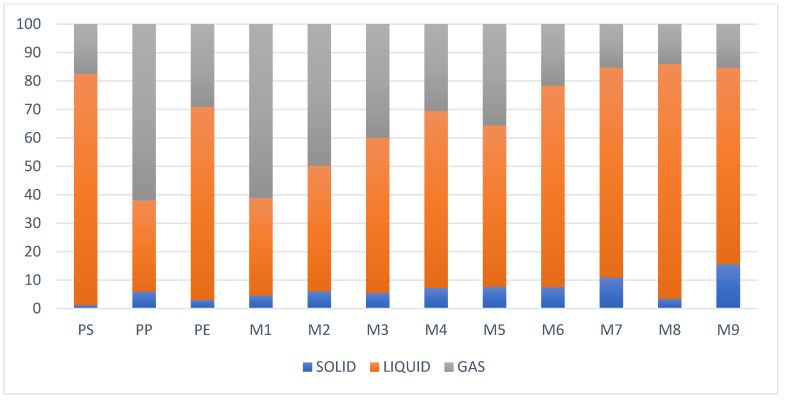
Yield of fractions, pyrolysis.

As mentioned above, the presence of PS in the pyrolysis process yields a fairly high liquid fraction close to 80%, while polyolefins such as PP and PE produce low liquid fraction yields, but with a high percentage of gaseous generation as shown in Figure 15. The liquid fraction from PP is the richest in light compounds before that obtained from PE, which contains a high percentage of waxes and rubbers, the same as those that occur in mixtures with PET. As for the liquid fraction obtained from the plastic mixtures, the presence of PS or a higher percentage of this plastic generates a yield greater than 60% in most cases, demonstrating better results with the mixture of 25% PP and 75% PS, being a mixture of hydrocarbons with liquid fractions close to 80% and with a composition between C6 and C20, a fairly low viscosity without affecting their caloric value.

Regarding the different mixtures tested, the mixtures M7, M8, and M9 are the ones that present a high yield due to the conjugation of PS and PP, while the mixtures M1, M2, and M3 present low yields compared to the previous ones due to the presence of PET that generates a higher gaseous fraction. While the mixtures M4, M5, and M6 have a significant liquid fraction, due to the presence of PE, they are mixtures that present gums and waxes highlighting among these the mixtures M3, M6, and M8 for their performance.

The values obtained for the liquid fraction of PE (68%) and PS (81%) are very similar to the values obtained in the studies of [8,40] at operating conditions between temperatures of 400 and 450 °C, where the values of PS produce high yields with the presence of styrene monomers, while the pyrolytic oil obtained from PE is presented with a very complex mixture of liquid and waxes [40,41]. The values of the liquid fraction obtained from PP (32%) are similar values to those shown by [9], and this low value compared to the gas production is attributed to the orientation to the formation of short chains by PP due to its methyl radical present in its structure and the random mechanism undergone by the pyrolysis of this plastic. With respect to the solid fraction, the studies presented by [8,36] show an amount of carbon between 1 and 8% very similar to the results obtained in this study. Regarding the mixtures, it is evident that the presence of polymers with oxygenated structures such as terephthalate orients the formation of gaseous products decreasing the liquid as in the case of mixtures between PP + PS and PET [26,35,41]. For mixtures with PE it can be observed that the presence of this polymer generates good performance, very close to that reported for plastic alone but with the presence of a higher percentage of wax when increasing the amount of PE [42].

### 3.7. Analysis of the Physicochemical Characteristics of the Pyrolytic Product

Table 3 presents the characteristics for the pyrolysis products of PP, PE, and PS, where it is observed that for PP and PE the pyrolysis products present a value of cetane index (27.6 and 29.15) greater than that presented by PS. (20.06), which is consistent with what is stated in the chromatography due to the presence of light and highly volatile compounds, a particular byproduct that indicates the high explosiveness when entering the cylinder of an engine that can generate greater noise in the engine and trigger emissions. However, at the moment of mixture there are mixtures that rise to about 50, and this is due to the interaction of the monomers present in the pyrolysis that generate more complex compounds and with a greater number of carbons, being of a greater contribution than those mixtures that present chains that are short in conjunction with an aromatic. Regarding the flash point, it presents a similar behavior in which individual plastic products have flash points at room temperature, while in mixtures they rise, reaching maximum values of 34 °C, which is still low for diesel standards, but which limit its use as a pure fuel. However, it could be considered as an additive in mixtures with fuels such as diesel or bunker, since in terms of its caloric power, all the results show values higher than 42 MJ per kilogram [36,42]. In addition, it was found that the sulfur content obtained is very low, complying with international regulations for its use as fuel in internal combustion engines ASTM D975.

On the other hand, in the liquid fraction of PS obtained in the best pyrolysis conditions, as shown in Table 3, the kinematic viscosity presents a value of 1.026 cSt (mm^2^ s^−1^) at 40 °C, which is less than that specified in the standard ASTM D975 for conventional diesel, in accordance with what is indicated by [26]. The low viscosity of the liquid fraction is attributed to the variations in the structure and composition of PS that differentiate it from other monomers, especially the presence of aromatic compounds, mainly styrene [41]. Favorable factors in fuel atomization are at low temperatures in burners [42]. The relative density at 15.6 °C of the liquid fraction obtained is 0.9352 g cm^−3^, which is similar to the values reported by [26]. The sulfur content of the liquid fraction obtained is low, which contributes as a fuel or additive to reduce the viscosity without the addition of sulfur in its structure. The caloric power of the liquid fraction has a value of 42,663 MJ kg^−1^. Several researchers have studied the liquid fraction of PS pyrolysis as an individual energy source or as a mixture with conventional diesel [42], due to its high amount of aromatic compounds that raise the flash point. However, there are other applications in which the main objective is the recovery of monomers, mainly styrene, and other compounds such as toluene and ethylbenzene that can be used as a chemical source in PS polymer polymerization industries [5].

On the other hand, the products obtained from PE are a mixture of hydrocarbons in a wide range that contain waxy substances, which generate specific properties of the pyrolytic product, as can be seen in Table 3, where the flash point obtained is quite low due to the presence of light compounds and a high viscosity with respect to the other fuels obtained from pyrolysis due to the presence of a large percentage of hydrocarbons with a chain greater than 20 carbons. The water content and the sulfur content are kept at low percentages, which is beneficial if this fuel will be used in engines or industrial applications [42,43,44]. Its main application according to its physicochemical properties would be as liquid wax.

Additionally, pyrolytic oils from plastic mixtures generate a mixture of many more compounds than those generated by individual plastics, corroborated by what is stated in [45]. In the M3 mixture that presents the highest yield of the liquid fraction, the presence of chain compounds with a high carbon content can be seen due to the pyrolysis of PET and PP, which is evidenced by a viscosity of 3.2 cSt, °API of 20.5 and a density of 930.9 kg m^−3^. Note that these are higher data than those presented by the pyrolysis of individual plastics. Additionally, the presence of PS in the pyrolysis causes aromatic and polyaromatic compounds to appear in the liquid fraction.

As for sample M6, it presented a yield of the liquid fraction of 70.1%, being the mixture with the highest liquid fraction without the presence of waxes, for which its properties were analyzed. These mixtures, as in the case of mixtures with PET, present a considerable amount of waxes or compounds with a high carbon number in their structure due to the presence of polyethylene; however, these samples have a lower density compared to the mixtures with PET, and a slightly lower viscosity that can be attributed to the greater amount of aromatic compounds or their derivatives, confirming a mixture of hydrocarbons with better performance the higher the percentage of polystyrene.

Finally, as can be seen in Table 3, the highest percentage of PS in the mixture (M8) provides a significant percentage of aromatics, resulting in a mixture of light hydrocarbons with low viscosities very close to 1 cSt, a flash point at room temperature (19 °C), and a low cetane rating, although it is a compound with a very high caloric value.

## 4. Conclusions

The overall analysis of the experiment shows the high potential for using plastics as a source of unconventional fuels, which closes the loop of the circular economy that can be envisaged for plastics. The physicochemical properties show that the presence of PS in the pyrolysis process produce a fairly high liquid fraction close to 80%, while polyolefins such as PP and PE produce low liquid fraction yields, but with a high percentage of gaseous generation. The liquid fraction from PP is the richest in light compounds before that obtained from PE, which contains a high percentage of waxes and rubbers, the same as those that occur in mixtures with PET. In the case of pure plastics, it is observed that at a temperature of 400 °C the performance of the liquid fraction is good both in quantity and quality. As for the mixtures of plastics, the presence of PS or a higher percentage of this plastic generates a performance greater than 60% in most cases, having better results with the M8 mixture, as it is a mixture of hydrocarbons with liquid fractions close to 80% and a composition between C6 and C20, and a viscosity of 1.12 cSt with a caloric power of 42.5 MJ per kilogram, which makes it a product with a high usage potential and a complement to the circular economy that is proposed with respect to plastic waste.

## Figures and Tables

**Table 1:** Operating conditions of the pyrolysis process.

Feature	Specification
Working Temperature	350–450 °C
Working Pressure	−0.05 MPa
Condensing Temperature	10 °C
Agitation	Low RPM
Heating Rate	12 a 15 °C min^−1^
Purge Gas	20 mL min^−1^ Nitrogen
Type of Plastic	PP, PS, PE and blends

**Table 2:** Physicochemical property parameters.

Parameter	Unit	Reference
Caloric Value	kJ kg^−1^	ASTM D-240
Distillation	°C	ASTM D86-18
Calculated Cetane	-	ASTM D4737
Flash Point	°C	ASTM D93
API at 60 °F	°API	ASTM D975
Specific Gravity (15.6 °C/15.6 °C)	-	ASTM D 287-92
Density at 15 °C	kg m^−3^	ASTM D1298
Sulfur Content	ppm	ASTM D4294
Kinematic Viscosity at 40 °C	cSt	ASTM D445
Water and Sediment content	% *v*/*v*	ASTM D1796

**Table 3:** Physicochemical characterization of the pyrolysis products.

Parameter	Unit	PP	PS	PE	M3	M6	M8
Caloric Power	kJ kg^−1^	47,103	42,663	46,490	44,751	44,479	42,515
Calculated Cetane	-	27.6	20.06	29.15	48.42	42.32	26.98
Flashpoint	°C	19	19	19	34	28	19
Specific Gravity (15 °C)	-	0.8343	0.8343	0.8124	0.9309	0.8893	0.8102
Density (15 °C)	kg m^−3^	833.9	833.9	812.4	930.9	889.3	810.2
Sulfur content	ppm	0.302	0.102	0.194	0.178	0.217	0.148
Kinematic Viscosity (40 °C)	cSt	1.60	1.03	1.70	3.20	2.70	1.12
Water and Sediment content	% *v*/*v*	0.60	0.35	0.39	0.27	0.25	0.28

## References

[1] Lazarevic D., Aoustin E., Buclet N., Brandt N. (2010). Plastic waste management in the context of a European recycling society: Comparing results and uncertainties in a life cycle perspective. Resour. Conserv. Recycl..

[2] Heidbreder L.M., Bablok I., Drews S., Menzel C. (2019). Tackling the plastic problem: A review on perceptions, behaviors, and interventions. Sci. Total Environ..

[3] Lee D.J., Lu J.S., Chang J.S. (2020). Pyrolysis synergy of municipal solid waste (MSW): A review. Bioresour. Technol..

[4] Wong S.L., Ngadi N., Abdullah T.A.T., Inuwa I.M. (2015). Current state and future prospects of plastic waste as source of fuel: A review. Renew. Sustain. Energy Rev..

[5] Lopez G., Artetxe M., Amutio M., Bilbao J., Olazar M. (2017). Thermochemical routes for the valorization of waste polyole fi nic plastics to produce fuels and chemicals. A review. Renew. Sustain. Energy Rev..

[6] Maqsood T., Dai J., Zhang Y., Guang M., Li B. (2021). Pyrolysis of plastic species: A review of resources and products. J. Anal. Appl. Pyrolysis..

[7] Honus S., Kumagai S., Fedorko G., Molnár V., Yoshioka T. (2018). Pyrolysis gases produced from individual and mixed PE, PP, PS, PVC, and PET—Part I: Production and physical properties. Fuel.

[8] Quesada L., Calero M., Martín-Lara M.Á., Pérez A., Blázquez G. (2020). Production of an Alternative Fuel by Pyrolysis of Plastic Wastes Mixtures. Energy Fuels.

[9] Miandad R., Barakat M.A., Aburiazaiza A.S., Rehan M., Ismail I.M.I., Nizami A.S. (2017). Effect of plastic waste types on pyrolysis liquid oil. Int. Biodeterior. Biodegrad..

[10] Solis M., Silveira S. (2020). Technologies for chemical recycling of household plastics—A technical review and TRL assessment. Waste Manag..

[11] Hujuri U., Ghoshal A.K., Gumma S. (2008). Modeling pyrolysis kinetics of plastic mixtures. Polym. Degrad. Stab..

[12] Abbas-Abadi M.S., Haghighi M.N., Yeganeh H., McDonald A.G. (2014). Evaluation of pyrolysis process parameters on polypropylene degradation products. J. Anal. Appl. Pyrolysis..

[13] Das P., Tiwari P. (2017). Thermal degradation kinetics of plastics and model selection. Thermochim. Acta..

[14] Xu F., Wang B., Yang D., Hao J., Qiao Y., Tian Y. (2018). Thermal degradation of typical plastics under high heating rate conditions by TG-FTIR: Pyrolysis behaviors and kinetic analysis. Energy Convers. Manag..

[15] Al-Salem S.M. (2019). Thermal pyrolysis of high density polyethylene (HDPE) in a novel fixed bed reactor system for the production of high value gasoline range hydrocarbons (HC). Process Saf. Environ. Prot..

[16] Meys R., Frick F., Westhues S., Sternberg A., Klankermayer J., Bardow A. (2020). Towards a circular economy for plastic packaging wastes—the environmental potential of chemical recycling. Resour. Conserv. Recycl..

[17] Mangesh V.L., Padmanabhan S., Tamizhdurai P., Ramesh A. (2020). Experimental investigation to identify the type of waste plastic pyrolysis oil suitable for conversion to diesel engine fuel. J. Clean. Prod..

[18] Singh R.K., Ruj B., Sadhukhan A.K., Gupta P. (2019). Thermal degradation of waste plastics under non-sweeping atmosphere: Part 1: Effect of temperature, product optimization, and degradation mechanism. J. Environ. Manage..

[19] Sogancioglu M., Ahmetli G., Yel E. (2017). A Comparative Study on Waste Plastics Pyrolysis Liquid Products Quantity and Energy Recovery Potential. Energy Procedia.

[20] Çepelioğullar Ö., Pütün A.E. (2014). Products characterization study of a slow pyrolysis of biomass-plastic mixtures in a fixed-bed reactor. J. Anal. Appl. Pyrolysis.

[21] Matias Á.A., Lima M.S., Pereira J., Pereira P., Barros R., Coelho J.F.J., Serra A.C. (2020). Use of recycled polypropylene/poly(ethylene terephthalate) blends to manufacture water pipes: An industrial scale study. Waste Manag..

[22] Singh N., Hui D., Singh R., Ahuja I.P.S., Feo L., Fraternali F. (2017). Recycling of plastic solid waste: A state of art review and future applications. Compos. Part B Eng..

[23] Nizami A.S., Rehan M., Ouda O.K.M., Shahzad K., Sadef Y., Iqbal T., Ismail I.M.I. (2015). An argument for developing waste-to-energy technologies in Saudi Arabia. Chem. Eng. Trans..

[24] Aisien E.T., Otuya I.C., Aisien F.A. (2021). Thermal and catalytic pyrolysis of waste polypropylene plastic using spent FCC catalyst. Environ. Technol. Innov..

[25] Torres J.M.A., Constante M.L.M., Borja E.O.P. (2014). Evaluación de la pirólisis térmica de aceite vegetal de desecho en un reactor batch. Revista Politécnica.

[26] Miandad R., Nizami A.S., Rehan M., Barakat M.A., Khan M.I., Mustafa A., Ismail I.M.I., Murphy J.D. (2016). Influence of temperature and reaction time on the conversion of polystyrene waste to pyrolysis liquid oil. Waste Manag..

[27] Nisar J., Ali G., Shah A., Iqbal M., Khan R.A., Sirajuddin, Anwar F., Ullah R., Akhter M.S. (2019). Fuel production from waste polystyrene via pyrolysis: Kinetics and products distribution. Waste Manag..

[28] Artetxe M., Lopez G., Amutio M., Barbarias I., Arregi A., Aguado R., Bilbao J., Olazar M. (2015). Styrene recovery from polystyrene by flash pyrolysis in a conical spouted bed reactor. Waste Manag..

[29] Zhou C., Yang Y., Li W., Shi Y., Jin L., Zhang Z., Wang G. (2018). Free radical reaction model for n-pentane pyrolysis. Chin. J. Chem. Eng..

[30] Sogancioglu M., Yel E., Ahmetli G. (2017). Investigation of the Effect of Polystyrene (PS) Waste Washing Process and Pyrolysis Temperature on (PS) Pyrolysis Product Quality. Energy Procedia.

[31] Mo Y., Zhao L., Wang Z., Chen C.L., Tan G.Y.A., Wang J.Y. (2014). Enhanced styrene recovery from waste polystyrene pyrolysis using response surface methodology coupled with Box-Behnken design. Waste Manag..

[32] Sharma S., Basu S., Shetti N.P., Kamali M., Walvekar P., Aminabhavi T.M. (2020). Waste-to-energy nexus: A sustainable development. Environ. Pollut..

[33] Baena-González J., Santamaria-Echart A., Aguirre J.L., González S. (2020). Chemical recycling of plastic waste: Bitumen, solvents, and polystyrene from pyrolysis oil. Waste Manag..

[34] Sharma B.K., Moser B.R., Vermillion K.E., Doll K.M., Rajagopalan N. (2014). Production, characterization and fuel properties of alternative diesel fuel from pyrolysis of waste plastic grocery bags. Fuel Process. Technol..

[35] Klaimy S., Lamonier J.F., Casetta M., Heymans S., Duquesne S. (2021). Recycling of plastic waste using flash pyrolysis—Effect of mixture composition. Polym. Degrad. Stab..

[36] Quesada L., Pérez A., Godoy V., Peula F.J., Calero M., Blázquez G. (2019). Optimization of the pyrolysis process of a plastic waste to obtain a liquid fuel using different mathematical models. Energy Convers. Manag..

[37] Singh T.S., Verma T.N., Singh H.N. (2020). A lab scale waste to energy conversion study for pyrolysis of plastic with and without catalyst: Engine emissions testing study. Fuel.

[38] Almeida D., Maria de Fátima M. (2015). Thermal and Catalytic Pyrolysis of Polyethylene Plastic Waste in Semi. Polimeros.

[39] Park K.B., Jeong Y.S., Guzelciftci B., Kim J.S. (2020). Two-stage pyrolysis of polystyrene: Pyrolysis oil as a source of fuels or benzene, toluene, ethylbenzene, and xylenes. Appl. Energy..

[40] Verma A., Sharma S., Pramanik H. (2021). Pyrolysis of waste expanded polystyrene and reduction of styrene via in-situ multiphase pyrolysis of product oil for the production of fuel range hydrocarbons. Waste Manag..

[41] Siddiqui M.N., Redhwi H.H. (2009). Pyrolysis of mixed plastics for the recovery of useful products. Fuel Process. Technol..

[42] Singh R.K., Ruj B., Sadhukhan A.K., Gupta P., Tigga V.P. (2020). Waste plastic to pyrolytic oil and its utilization in CI engine: Performance analysis and combustion characteristics. Fuel.

[43] Singh R.K., Ruj B., Sadhukhan A.K., Gupta P. (2019). Impact of fast and slow pyrolysis on the degradation of mixed plastic waste: Product yield analysis and their characterization. J. Energy Inst..

[44] Mani M., Nagarajan G., Sampath S. (2011). Characterisation and effect of using waste plastic oil and diesel fuel blends in compression ignition engine. Energy.

[45] Miandad R., Barakat M.A., Rehan M., Aburiazaiza A.S., Ismail I.M.I., Nizami A.S. (2017). Plastic waste to liquid oil through catalytic pyrolysis using natural and synthetic zeolite catalysts. Waste Manag..

